# Disparities in Testicular Cancer: A Review of the Literature

**DOI:** 10.3390/cancers16203433

**Published:** 2024-10-10

**Authors:** Domenique Escobar, Siamak Daneshmand

**Affiliations:** Catherine and Joseph Aresty Department of Urology, University of Southern California/Norris Comprehensive Cancer Center, University of Southern California, 1441 Eastlake Ave. NOR 7416, Los Angeles, CA 90033-9178, USA; daneshma@med.usc.edu

**Keywords:** testicular cancer, testis cancer, health disparities, cancer outcomes, socioeconomic status

## Abstract

**Simple Summary:**

In this study, we wanted to better understand the differences that exist between different groups of patients with testicular cancer, such as those from various racial/ethnic backgrounds, states and countries, ages, gender identities, insurance statuses, and financial statuses, amongst others. We were interested in looking at differences in these patients—including incidence, death rates, treatments and more. In our own clinical practice, we see how certain groups of patients present later, with worse disease, or die more often than others, and we feel it is critical to understand the research that exists in this space. We hope to raise awareness on the many disparities that exist and encourage further research and solutions.

**Abstract:**

**Background**: Testicular cancer is the most common malignancy diagnosed in adolescents and young adults, and evidence has emerged regarding disparities that affect different groups of patients. **Methods**: In this article, we conducted a thorough review of this area and summarized the existing literature. **Results**: Some of the pertinent findings from our review include poorer outcomes for various groups including the native Māori population of New Zealand, those who live in the United States–Mexico border region, those who live in Eastern Europe, those who are uninsured and those with poorer socioeconomic status, amongst others. In the United States specifically, there is significant evidence showing that racial/ethnic minorities, compared to white patients, tend to fare worse with later presentation at higher stages and worse survival rates. Hispanic patients in particular appear to have the potential for more aggressive tumor biology than other groups and are projected to have the highest incidence rates in the US by 2026. **Conclusions**: Overall, disparities exist in many aspects of testicular cancer and are striking in some instances, and further research is needed in this arena and in potential solutions.

## 1. Introduction

Testicular cancer is the most commonly diagnosed cancer in adolescents and young adults in the United States (US) [1] and represents a wide clinical spectrum. Testicular cancer includes germ cell tumors (both seminoma and non-seminoma [NSGCT]) and rarer tumors such as Sertoli cell and Leydig cell tumors, gonadoblastomas and lymphomas. Fortunately, 5-year survival is excellent at 95%, according to the National Cancer Institute Surveillance Epidemiology and End Results Program (SEER).

Incidence is rising in all racial/ethnic groups in the US [2,3] but Hispanic individuals are projected to have the highest rates of all groups by 2026 [4]. Incidence rates in Hispanic patients in California interestingly appear to be higher in higher socioeconomic status (SES) groups, although with significant incidence increases in low-SES groups [5]. Worldwide, incidence has also increased over time, and while mortality has remained stable, trends have varied widely between countries and regions of the world [6,7,8,9].

The etiologies of testicular cancer are varied, but one of the strongest risk factors is cryptorchidism [10], and seminoma is the most common histology in this setting. Interestingly, there is some evidence that patients with cancer in an undescended testicle are more likely to be from a racial/ethnic minority group or foreign-born [11].

Disparities exist in various aspects of testicular cancer and remain poorly understood. A recent review of early-stage testicular cancer found that lower income, Medicaid or uninsured status, minority race/ethnicity, and community setting of care were all associated with poorer outcomes in certain patients [12]. Another review that evaluated socioeconomic status (SES) found mixed associations regarding the development of testicular cancer, that lower educational levels and higher poverty levels were associated with later stage of diagnosis, and that lower SES was associated with increased mortality [13]. On the other hand, another study found that testicular cancer was associated with lower poverty rates [14]. One study uniquely evaluated testicular cancer perceptions amongst African American and white college students and found that African American men had less knowledge about testicular cancer, more cancer fatalism and perceived themselves to be of lower risk of the disease [15]. A case report of an African American man presenting with widely metastatic seminoma highlights some of the these factors that may contribute to disparities including but not limited to emotions such as fear and anxiety, variations in education level, financial and insurance issues, and distrust of the medical system [16].

The purpose of this review is to thoroughly evaluate and summarize the existing literature regarding disparities in testicular cancer.

## 2. Materials and Methods

**Figure 1:**
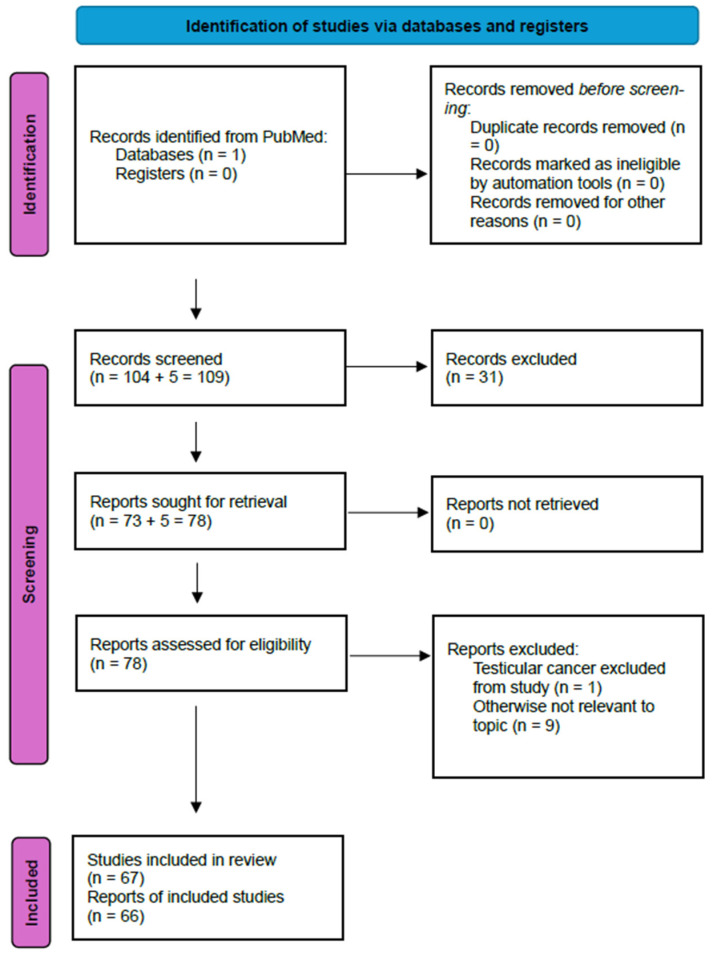
PRISMA diagram for article selection. PRISMA diagram detailing process for identification and inclusion of articles into review.

A PubMed literature search was conducted using the search term “testicular cancer disparities”, which resulted in 104 articles. Appropriate articles were selected for inclusion by reviewing article titles and abstracts. Additional searches were performed using the search terms “testicular cancer health disparities”, “testis cancer disparities” and “testis cancer health disparities”, and 5 additional articles were identified, all of which were included. Lastly, pertinent articles found from references from the previously identified articles were also included. Two additional articles were included from this method. Appropriate articles were initially screened for inclusion by reviewing article titles and abstracts. Articles that did not include a focus on testicular cancer or that were otherwise irrelevant were excluded. After this initial screening was completed, the full text of the remaining articles was reviewed, and articles that excluded testicular cancer or were otherwise irrelevant were excluded. The remaining articles that were included all included data on various aspects of disparities in testicular cancer. PRISMA guidelines were followed for article selection. This study has not been registered. Articles were separately reviewed by both authors for suitability and one author collected the data for inclusion. The PRISMA diagram for article selection is shown in Figure 1.

## 3. Results

### 3.1. Biological Etiologies

One study sought to better understand the lower incidence of testicular cancer in men of African ancestry and evaluated previously reported single nucleotide polymorphisms (SNPs) significantly associated with testicular cancer. A total of 79 risk SNPs were identified, and the average risk allele frequencies were found to be significantly lower in men of African ancestry than men of European ancestry. Interestingly, three of the four outlier SNPs are located in the KITLG gene, which has been shown to be involved in the development of testicular tumors [17].

### 3.2. Worldwide Trends

**Figure 2:**
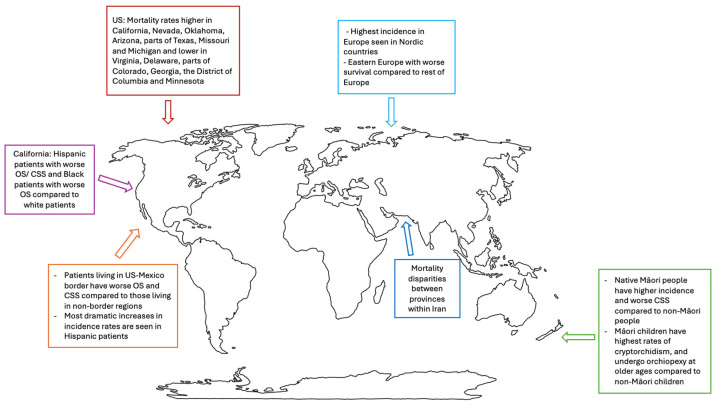
World map highlighting notable testicular cancer disparities findings specific to geography.

A fair amount of evidence exists regarding trends and disparities in testicular cancer worldwide. Here, we will highlight areas that have been most well studied. A summary of some of the most notable findings specific to geography is shown in Figure 2.

### 3.3. New Zealand

Multiple studies have described the testicular cancer disparities that exist for the Native Māori population of New Zealand, a country that has a diverse population and has also seen rising incidence of testicular cancer over time [18]. For example, Māori people have been shown to have a poorer overall 5-year cancer-specific survival (CSS), and that this is most pronounced in adolescents [19], significantly greater incidence of disease compared to non-Māori people [19,20] and appear to present at younger ages and with more advanced disease (for NSGCT only) [21]. While one study showed worse CSS in Māori people [20], another study found that overall survival (OS) is similar within each risk group, regardless of ethnicity [21]. One study also identified an interesting divergence between Māori people and Pacific Island New Zealanders (those who live in New Zealand but identify as belonging to the ethnic group of one or more Pacific Island (e.g., Samoa, Tonga, Cook Islands)), where rates of disease are much higher in the Māori people. This is in contrast to the majority of other diseases where rates are similar between the two groups [22,23]. Some of the potential etiologies identified for this divergence include higher rates of smoking in pregnant Māori women, higher rates of low birth weight, short gestational duration and small size for gestational age in Māori infants, and relatively high rates of cannabis use and smoking amongst Māori people. Māori children have also been found to have the highest rates of cryptorchidism compared to all other ethnic groups [22] and appear to undergo orchiopexy at older ages compared to non-Māori children [24], which may in part explain some of these differences. In contrast to the much of the literature where higher incomes are associated with higher rates of testicular cancer, one study found that men in New Zealand with low incomes had an increased risk of testicular cancer compared to those with high incomes, after adjusting for ethnicity [18].

### 3.4. Mexico

Testicular cancer is a significant health concern in Mexico, and the country has one of the highest incidences of testicular cancer in the Latin American Caribbean region [25]. One study aimed to explore the association between various sociodemographic factors in Hispanic men with testicular cancer and found that significant risk factors for mortality included NSGCT, advanced stage, younger age and lower education level. Survival time was significantly affected by lower SES and higher education. Patients with NSGCT and lower SES had significantly worse OS and higher education level, which positively impacted treatment response [25]. Another study looked specifically at patients in US–Mexico border regions (using SEER and the Texas Cancer Registry—although it is important to note that Arizona and undocumented individuals are not included in SEER) and found that patients with testicular cancer living in the border region had worse OS and that incidence rates in Hispanic patients in all regions are increasing at a significantly higher rate than non-Hispanic white patients. More advanced stage at diagnosis was also seen in at higher rates in Hispanic patients compared to non-Hispanic white patients in non-border states, non-border counties and in border counties. Patients in border counties also had significantly worse CSS compared to non-border states, regardless of race/ethnicity. There are many potential contributing factors to this finding in the border region including differences in access to care, language barriers, lower education levels and SES, higher rates of being uninsured, and high rates of poverty. In addition, some border states, Texas and New Mexico in particular, experience very low numbers of urologists (~3) per 100,000 persons, further complicating access to urologic care specifically. Interestingly, the disparities in CSS were only seen in testicular cancer and none of the other urologic malignancies. Other important factors that may explain this finding include environmental factors including pesticide and heavy metal use (high rates of agriculture in some border regions) and higher rates of NSGCT in some border regions [26]. Other studies have similarly shown high rates of mortality in border regions [27].

### 3.5. Europe

Drastic geographical disparities have also been noted in Europe. Nordic countries, for example, have been shown to have the highest incidence rates, while Eastern European countries have the highest mortality rates, compared to the rest of Europe [28]. Another study similarly found that the 5-year relative survival in Eastern Europe was 88%, compared to >95% for all other regions of Europe [29]. One study out of Geneva, Switzerland, a, country with relatively high rates of testicular cancer, found that patients with NSGCT and patients with low SES were more often diagnosed at advanced stages and that SES was significantly associated with 10-year OS and CSS [30]. In contrast to the majority of the literature, one study from the United Kingdom found no impact of SES on survival when assessing two studies—one with patients with stage I NSGCT and one with patients with stage I seminoma [31].

### 3.6. United States (US)

Many studies have been performed in the US to describe disparities in testicular cancer, commonly using major databases and registries.

### 3.7. SEER

SEER data have been utilized in several studies to evaluate disparities in incidence, treatments and outcomes in the US. Although it is important to note that SEER does not fully capture all cases or sites and does not perfectly represent the US population, the data have still allowed for important observations. For example, white patients have been found to have the highest incidence rates (followed by Hispanic, Asian/Pacific Islander and Black patients) and the highest survival rates (compared to Black patients who have the lowest survival rates) [32,33,34]. Asian Americans, when compared to white patients, have also been found to present with higher stage and with higher rates of seminoma [35]. Older data have also shown that compared to white patients, African American, Native American, Asian/Pacific Islander and Hispanic patients were more likely to be diagnosed at later stages [36,37,38] and more likely to die of the disease [37,39]. Hispanic men in particular have been shown to be diagnosed at the youngest age amongst all groups [38,40], with a longer delay to diagnosis, with a significantly larger tumor size at presentation and to be more likely to have NSGCT histology [40]. Coupled with being diagnosed at later stages, indicates the potential for more aggressive tumor biology in Hispanic patients.

Another study utilizing SEER found that in contrast to much of the literature, incidence rates were highest in American Indian and Alaska Native patients, but more consistent with the literature, that incidence was lowest in Black patients, and mortality rates increased in the Hispanic population but decreased in the white population [2].

Other studies utilizing SEER to evaluate factors associated with metastatic disease found that younger patients, racial/ethnic minorities (Hispanic, Black [33,39] or American Indian/Alaskan), single or separated patients, uninsured patients and patients of lower SES [39] were significantly more likely to present with metastases at diagnosis. In multivariable analysis, predictors of metastases at diagnosis similarly included older age, Southeastern US residence, American Indian/Alaskan race, Hispanic ethnicity, lower SES, and single marital status [41].

Another study, also utilizing SEER, looked specifically at outcomes in children and adolescents with germ cell tumors (both gonadal and extra-gonadal) and found that compared to white patients, Asian/Pacific Islander and Hispanic patients had significantly higher risks of death and that this association was mediated by stage of disease only in Hispanic patients with gonadal tumors. Black and Hispanic patients also had the highest proportions of distant disease [42]. Adolescents have also been shown to have poorer outcomes compared to children and are often under-represented in clinical trials [43]. Specifically, when looking at adolescent and young adults in California, Hispanic patients had worse OS and CSS and Black patients had worse OS compared to white patients, after adjusting for neighborhood SES. This was most pronounced in NSGCT as compared to seminoma. Residing in a low- or middle-SES neighborhood was also significantly associated with worse OS and CSS, even after adjusting for race/ethnicity [44].

Other studies have found drastic differences in mortality outcomes between individual counties and states in the US. For example, the rates of change in mortality ranges from a decrease of 72% in Nantucket County, Massachusetts to an increase of 39% in Union County, Florida. Mortality rates were also shown to be higher in California, Nevada and parts of Texas, Missouri and Michigan and lower in parts of Colorado, Georgia, the District of Columbia and Minnesota [27].

### 3.8. NCDB

The National Cancer Database (NCDB) has also been used to examine these relationships. Studies have similarly shown that Black race or Hispanic ethnicity and low SES were associated with diagnosis at later stage [45] and poorer outcomes [46].

One study looked specifically at patients at a tertiary care cancer center in New Mexico, a uniquely vulnerable state due to very low numbers of urologists, majority Hispanic population, variability in health literacy, highest rates of Medicaid enrollment, and being a rural state with need for significant travel distances to access care. Notably, this study found that Hispanic and Native American patients had longer delays to initial presentation, presented at higher stages and that in stage II disease, Hispanic patients were significantly less likely to receive RPLND compared to white patients. Interestingly, amongst all patients with stage II disease, rates of RPLND in New Mexico were significantly lower than national rates (30% vs. 58%). In stage I disease, white patients were also significantly more likely to receive chemotherapy whereas Hispanic patients were more likely to not undergo adjuvant therapies, especially when they lacked insurance. Hispanic patients were found to have higher rates of nonadherence (defined as missing three or more appointments or being lost to follow up), specifically in stage II/III disease [47]. A similar study out of Florida showed that in stage I NSGCT, patients with a college education or higher or those who lived closer to the medical center were more likely to choose surveillance [48].

One study sought to assess the impact of rurality and urologist density on outcomes in patients with NSGCT and found that RPLND was performed more often in patients who lived in a county with a urologist and in an urban setting. In addition, the number of lymph nodes examined was lower in rural counties and in counties with fewer urologists. However, the number of positive lymph nodes or rates of cancer-specific mortality was not associated with rurality or urologist density. Black patients also had more than double the risk of cancer-specific mortality compared to white patients, and education level was negatively correlated with cancer-specific mortality [49]. Another study found that significant differences in incidence rates in rural and urban settings were present amongst different racial/ethnic groups—the rates for White/Caucasian and Hispanic people were higher in urban regions, whereas rates for American Indian/Alaskan Natives and Asian/Pacific Islanders were higher in rural regions. This study also found that incidence rates of stage II disease were significantly higher in urban areas than rural ones. Mortality rates, however, appeared to be similar between urban and rural settings [50].

Little research has been undertaken to explore trends in testicular cancer in the Native populations of the US, and it is important to note that incidence rates may be underestimated due to racial misclassification in this group [51] and relatively low capture rates in national databases [52]. One study looked at incidence rates and trends in various cancers in Non-Hispanic American Indian and Alaska Native adolescents and young adults and found that, as expected, testicular cancer had the highest incidence of all cancers in males in all regions (although notably in the East only, rates were lower than that of the US overall rates) and in both age groups. Testicular cancer also accounted for nearly a quarter of all new cancers in this population [53].

### 3.9. CDC-WONDER

One study using data from the CDC-WONDER database from 1999–2020 found that in testicular cancer, age-adjusted mortality rates increased significantly in white patients, remained stable in Black and African American patients and increased in non-metropolitan areas. The highest rates were observed in the West, Midwest, Northeast, and South in descending order. Specifically, the highest mortality rates were seen in Oklahoma, followed by Arizona and California, whereas Virginia and Delaware had the lowest rates [54]. A similar study also utilizing CDC-WONDER (and NAACCR) over the same time period found that in Hispanic patients, both incidence of metastatic disease and cancer-specific mortality significantly increased, and that Black and Hispanic men were more likely to die in medical facilities [55]. Although relative survival has increased for all groups over time, it has been shown to be lower in Black and Hispanic men compared to white men, in addition to being lower in high-poverty neighborhoods [56].

### 3.10. Histology-Specific Outcomes

#### 3.10.1. Non-Seminoma

There is growing evidence highlighting the disparities that exist in outcomes based on histology. In general, NSGCT confers a higher mortality rate compared to seminoma [34]. Recent studies have also shown that OS differs by racial/ethnic group. One study of patients with stage III NSGCT found that compared to simulated age-matched controls, OS rate differences were largest in Asian/Pacific Islander patients (difference of 35%), followed by African American (31%), Hispanic (27%), and Caucasian people (22%). This study also found that the 5-year CSM was highest in Asian/Pacific Islanders (32%), followed by African American (26%), Hispanic (25%), and Caucasian people (20%) [57]. Another study of over 13,000 patients evaluated CSM across stages and found that in all patients, CSM was higher in non-Caucasian patients compared to Caucasian people and that specifically, race/ethnicity was an independent predictor of CSM in Hispanic people in stages I–III, in African American people in stage I and III and in Asian people in only stage III [58]. In stage II disease, patients of lower SES, Black race or who are unmarried have also been found to be less likely to receive RPLND and to have higher risks of cancer-specific mortality [59]. Some studies have also shown differences in treatment based on provider specialty. One study found that in stage I NSGCT, rates of RPLND were significantly higher if the patient was evaluated by a urologic oncologist as opposed to a medical oncologist or multidisciplinary team. However, rates of surveillance in this setting were equivalent despite provider type [48].

#### 3.10.2. Seminoma

One study utilizing the NCDB looked at trends in management of stage I seminoma and how social and demographic factors may affect choice of therapy. Surveillance after orchiectomy in stage IA/B, which has become the preferred option according to the National Comprehensive Cancer Network (NCCN), was more common in racial minorities compared to white patients and in uninsured or Medicare patients compared to privately insured patients. However, the authors note that it is possible that, even though it appears that these groups are receiving the preferred care, these patients may actually be receiving substandard care due to lack of follow up or poorer access to medical care. Surveillance was also less common in non-academic centers, in facilities with lower seminoma volume and in patients with T2 or T3 disease or tumors 4 cm or larger [60].

### 3.11. Fertility Preservation

There is evidence that disparities exist with regard to fertility preservation, although the effects appear to be somewhat mixed. For example, one study in the US compared the use of sperm cryopreservation between patients at a private tertiary care academic center and an affiliated public safety-net hospital and found that treatment at the academic center was strongly associated with use of cryopreservation [61]. A study completed in the United Kingdom (where there are no standardized funded fertility preservation services for children with cancer), surveyed all 20 pediatric cancer service centers and found geographical differences in referrals for testicular tissue storage as well as wide variation in funding sources (charitable vs. research vs. National Health Service) [62]. Although this study looked at patients with all forms of cancer, similar findings would likely be expected for testicular cancer which is one of the most common cancers in adolescents and young adults. Another study of four cancer centers in the US sought to assess the rates of documentation of discussion of risks of infertility, fertility preservation options, and referral to a fertility specialist in adolescent and young adult patients. This study evaluated 283 total patients with the most common cancer types in this age group (breast, leukemia/lymphoma, sarcoma, and testicular), 31 of whom had testicular cancer. Interestingly, documentation of discussion of risk of infertility risk was significantly more likely in patients with sarcoma or testicular cancer (as compared to patients with breast cancer), as was documentation of discussion of fertility preservation options [63].

### 3.12. Lesbian, Gay, Bisexual, Transgender and QUEER + (LGBTQ+) Community

There is a growing population of lesbian, gay, bisexual, transgender, queer and other sexual and gender minorities (LGBTQ+) both in the US and worldwide [64] and incidences of cancer in the transgender population are likely underestimated [65]. Thus, it is critical for providers to be equipped to care for this population and to understand the barriers that exist for them. For example, transgender and gender-diverse people are more likely to experience microaggressions, discrimination, negative healthcare experiences and various social stressors [66]. In addition, they may have variable levels of identifying or associating with their genitalia, which may in turn affect important health care examinations such as testicular, penile or prostate exams. These factors, in addition to many others, can result in healthcare avoidance and potentially poorer outcomes. The risk of testicular cancer in transgender women appears to be equivalent to that of cisgender men and does not seem to be impacted by the use of hormonal therapy [67]. The practice of “tucking” to reduce the appearance of a genital bulge, however, has been shown to increase the risk of testicular cancer over time, thought to be due to testicular position being similar to that of an undescended testicle [68]. A recent case report of a 47-year-old transgender woman with a history of testicular cancer who presented 20 years later with a 15 cm metastatic neck mass highlights many of the barriers trans people face, including insurance, financial, transportation difficulties, and many of the reasons for delayed presentations to care, including gender dysphoria and disassociation with genitalia [69].

### 3.13. Insurance

Various studies have looked at the effects of insurance status and outcomes in testicular cancer. A study from New Mexico found that while insurance rates did not differ between Hispanic and white patients, insurance did increase the likelihood of receiving chemotherapy in stage I and RPLND in stage II/III for white patients only and that lack of insurance increased rates of surveillance only in Hispanic patients with stage I disease [47]. In this study, after implementation of the Affordable Care Act (ACA), insurance rates did not appear to change but the incidence of testicular cancer increased 58% amongst Hispanic patients (compared to 8% in white patients). Other studies utilizing NCDB have evaluated patients with metastatic disease and found that insurance rates did not change and metastatic presentation increased after the ACA was enacted, and that predictors for presenting with metastatic disease were Medicaid, Medicare and uninsured status (compared to privately insured) [70]. Underinsurance has been shown to be significantly associated with poor outcomes [46]. Another study similarly found that patients without insurance or with Medicaid or Medicare were more likely to be diagnosed at later stages [45].

Other studies utilizing SEER data have similarly shown that both uninsured and patients with Medicaid were more likely to present with a larger tumor, be diagnosed with advanced disease (both metastatic and intermediate/poor-risk disease), less likely to undergo RPLND [59], less likely to receive radiation in stage I seminoma (for uninsured patients only), and more likely to die from their disease [41,71]. Relative survival has also been shown to be poorer in patients with Medicaid compared to those with private insurance [56]. Similar findings have been shown in Ohio specifically, in which there are significant survival differences in patients with Medicaid compared to private insurance and in addition, differences based on timing of enrollment into Medicaid [72]. When compared to countries with public insurance (such as the UK and Germany), 10-year CSS has been found to be significantly lower in the US, where it is not uncommon for young men to be uninsured [73].

## 4. Discussion

While the disparities in testicular cancer are broad, multi-dimensional and tremendous in some instances, they do highlight various opportunities to close these gaps and improve outcomes for the most vulnerable populations. In this review, we highlighted many groups that are affected by disparities including racial/ethnic minorities, adolescents, those who are uninsured, those who are single, transgender individuals, and more. This is summarized in Table 1. It is critical that measures to address these disparities take into account the diversity that exists amongst patients with testicular cancer. There is evidence for significant differences in outcomes between high- and low-volume centers in patients with poor-risk metastatic disease, also highlighting the importance of centers of excellence in caring for these patients [46,74].

While the benefits of testicular self-examination are unclear (and screening for testicular cancer has not been recommended by the US Preventive Service Task Force) encouraging broader discussion and increasing awareness of testicular cancer are potential mechanisms to improve diagnosis and outcomes, particularly in some of the more at-risk groups such as adolescents or African Americans [75]. In the US, it is also important to be aware of resources that already exist. For example, Planned Parenthood offers testicular cancer screening and referral services, although availability of these services can be variable and some groups (particularly Black and African American men) may have difficulty accessing these services [76].

Many patients turn to the Internet to gather information about medical conditions, including cancer. Spanish-speakers are increasingly using the Internet and interestingly, testicular cancer, in particular, is searched more frequently by Spanish-speakers compared to non-Spanish-speakers [77]. Ensuring there is accurate information in a patient’s native language is another way to ameliorate some of the gaps.

In Ireland, a focus group composed of multiple stakeholders identified important components of a community-based testicular cancer awareness campaign including the use of social media, posters, radio and television advertisements and with a focus on behavioral targeting and education (i.e., overcoming embarrassment) and being inclusive of various groups (age, gender identities and sexual orientations) [78]. The same group also previously highlighted the importance of taking into account young men’s preferred learning strategies [79]. Many of these factors should be considered in patient outreach. Overall, further research is needed to better understand the many disparities that exist and into their etiologies and potential solutions.

## 5. Conclusions

Disparities exist across the spectrum in testicular cancer, including time to presentation, diagnosis, treatments, and survival. Differences exist worldwide and racial/ethnic minorities, adolescents and those who are of lower SES or uninsured appear to be the most impacted. More research is needed for further elucidate the etiologies of the various disparities identified, as well as to highlight potential future directions to ameliorate them.

## Figures and Tables

**Table 1:** Summary of major risk factors for testicular cancer incidence/development and mortality.

Incidence/Development of Testicular Cancer	Mortality
Cryptorchidism Certain single nucleotide polymorphisms (SNPs) Native Māori population of New Zealand, compared to non-Native population and Pacific Island New Zealanders Higher income (data mixed) US: White race, Hispanic ethnicity Testicular “tucking”	Native Māori population of New Zealand (data mixed) Mexico: NSGCT, advanced stage, younger age, lower education level, lower SES Residing in the US–Mexico border region Europe: residing in Eastern Europe, lower SES (data mixed) US: racial/ethnic minorities (Black, Hispanic, Native American, Asian/Pacific Islander), lower SES, lower education levels, residing in certain states (OK, AZ, CA), unmarried status, lack of insurance or Medicaid insurance Non-seminoma histology
